# Construction of Biocompatible Dual-Drug Loaded Complicated Nanoparticles for *in vivo* Improvement of Synergistic Chemotherapy in Esophageal Cancer

**DOI:** 10.3389/fonc.2020.00622

**Published:** 2020-05-05

**Authors:** Wenhua Zhan, Hanrui Li, Yingying Guo, Getao Du, Yayan Wu, Dexin Zhang

**Affiliations:** ^1^Key Laboratory of Biomedical Information Engineering of Education Ministry, School of Life Science and Technology, Xi'an Jiaotong University, Xi'an, China; ^2^Department of Radiation Oncology, General Hospital of Ningxia Medical University, Yinchuan, China; ^3^Engineering Research Center of Molecular & Neuro Imaging of the Ministry of Education, School of Life Science and Technology, Xidian University, Xi'an, China; ^4^Department of Respiratory Medicine, Second Affiliated Hospital of Medical College, Xi'an Jiaotong University, Xi'an, China

**Keywords:** esophageal cancer, combination chemotherapy, doxorubicin, β-elemene, mesoporous silica nanoparticles

## Abstract

Combination chemotherapy is a routine treatment for esophageal cancer, but some shortcomings, such as drug toxicity and side effects, greatly limit the clinical application of combination therapy. To overcome these shortcomings, we have developed a mesoporous silica nanoparticle system that was used to load doxorubicin and β-elemene. β-elemene was encapsulated in the pore of mesoporous silica nanoparticle and doxorubicin was electrostatically adsorbed on the surface of mesoporous silica nanoparticle by hyaluronic acid to construct dual drugs synergistic nanoparticles (bMED NPs, ~77.15 nm). *In vitro* studies demonstrated that bMED NPs had a good treatment effect in esophageal cancer cell lines. *In vivo* fluorescence imaging results demonstrated that bMED NPs could accumulate in tumor sites and achieve *in vivo* long-term circulation and continuous drug release. In addition, bMED NPs exhibited significant antitumor effects in the esophageal cancer mouse model, which may provide a great platform for esophageal cancer chemotherapy.

## Introduction

Esophageal cancer is the sixth leading cause of cancer deaths in the world (1). The incidence is higher in East Asia, southeastern Africa and northwestern Europe. In China, esophageal cancer is also one of the four major cancers (2). Most cases of esophageal cancer are diagnosed at an advanced stage and the 5-year survival rate is very poor (3). Surgery has a certain therapeutic effect in the clinic, however, surgery-caused damage is irreversible, and the patient quality of life is greatly reduced. Meanwhile, surgery may be followed by cancer recurrence and tumor metastasis (4, 5). Combination chemotherapy is a common method for the treatment of esophageal cancer and when compared with single drug treatment, this approach has an obvious superiority (6). By using several drugs, the possibility of a significant increase in single drug toxicity caused by drug overdosing can be avoided (7, 8). In addition, the long-term and repeated use of the same drug is one of the factors that lead to tumor resistance. Combination chemotherapy can prevent resistance factors, thereby reducing the possibility of cancer resistance (9).

The choice of drugs is critical for combination chemotherapy. In previous studies, many chemotherapeutic drugs were used in esophageal cancer therapy. Among them, cetuximab is one of the FDA-approved chemotherapeutic drugs that has a certain therapeutic effect on esophageal cancer (10). However, in previous reports, cetuximab has a marked increase of toxicity on 75 years of age or older patients and in 2011, DMC and NCI approved an amendment to limit cetuximab treatment to the under 75 years of age (11). Doxorubicin (DOX) is a commonly used chemotherapy drug for cancer (12, 13) and DOX is also widely used in the treatment of esophageal cancer (14, 15). However, DOX has an inevitable cardiotoxicity and severe side effects, resulting in a poor prognosis (16). There are many studies on the combination of DOX and other anticancer drugs for esophageal cancer that aim at improving the antitumor effect (17, 18). However, due to the differences in the metabolic timing, pathways and patterns of the two drugs, it was difficult to determine the optimal dose and ratio of the drug to exert optimal therapeutic effects at the tumor sites. Therefore, the drugs optimal ratio is critical for combination chemotherapy, and a more effective combination is essential. β-elemene is a natural drug that was shown to exert antitumor effects in a variety of tumors (19–23). It is mainly used as an adjuvant in cancer treatment to improve the efficacy of chemotherapy, reduce its toxicity and prevent drug resistance (24). In previous studies, it has been shown that DOX and β-elemene can be used in combination therapy for tumors (25). In addition, previous studies showed that the insertion of DOX into duplex DNA is the main reason for its anti-cancer activity through the inhibition of DNA replication (26), while, β-elemene may exert its antitumor effect by mediating via a mitochondrial cytochrome c release-dependent apoptotic pathway and downregulating the expression of Bcl-2 (27). Therefore, DOX and β-elemene may be able to cause apoptosis through different signaling pathways, which is of great significance for tumor combination chemotherapy.

Our previous studies demonstrated that DOX and β-elemene had synergistic effects. However, the physical and chemical properties of DOX and β-elemene are different, with *in vivo* differences in pharmacokinetics, which lead to decreased in accumulation, in an optimal ratio and with a certain concentration *in vivo*. Nanocarriers can load two or more chemotherapeutic drugs in an optimal ratio and achieve better therapeutic effects. In previous studies, a variety of nanocarriers have been reported as an option for combination therapy, such as micelle (28), nanoparticle (29, 30) and vesicular nanocarriers (31). Among these, the mesoporous silica nanoparticle has a large specific surface area and pore size, low toxicity and a surface which can be easily modified for common use as a nanocarrier (32).

To study the combination therapeutic effects of β-elemene and DOX on tumors, we synthesized biodegradable mesoporous silica nanoparticles (bMSN NPs) as nanocarrier for the dual-drug combination therapy and according to the previously described method (33). β-elemene is adsorbed in bMSN pores and the DOX surface is covered with hyaluronic acid (HA) using electrostatic adsorption. Both β-elemene and DOX are loaded on the bMSN NPs in an optimal ratio (bMED NPs) to improve the efficacy of the dual-drug nanoparticles. The bMED NPs based on the combination chemotherapy effects of β-elemene and DOX, can enhance their pharmacokinetic characteristics and promote their passive targeting via enhanced permeability and retention (EPR) effects, thereby, reducing their toxicity. The bMED NPs have been proven to have low toxicity, good biocompatibility and good targeting capabilities through a series of *in vitro* experiments. Furthermore, to verify the antitumor effect of bMED NPs *in vivo*, a subcutaneous tumor model of esophageal cancer was established, and the mice body weight and tumors size were monitored for 22 days. The results indicated that the synthesized bMED NPs exert excellent antitumor effects *in vitro* and *in vivo* and provide a novel drug choice for the treatment of esophageal cancer.

## Materials and Methods

### Materials

β-elemene were purchased from Sigma. DOX (98%), HA, hyaluronidase (HAase), coumarin 6, crystal violet, Giemsa stain, 4′,6-diamidino-2-phenylindole (DAPI), propidium iodide (PI), annexin V-100 and triton X-100 were purchased from Aladdin (Shanghai, China). IR780 iodide (95%) were purchased from Sigma-Aldrich. Fetal Bovine Serum (FBS) was purchased from Biological Industries (Israel). Dulbecco's modified eagle medium (DMEM) was purchased from Hyclone. Cell Counting Kit-8 (CCK-8) was purchased from Dojindo (Shanghai, China). Terminal deoxynucleotidyl transferase-mediated dUTP-biotin nick-end labeling (TUNEL) Apoptosis Detection Kit were purchased from Beyotime (Shanghai, China). The primary anti-Bcl-2, anti β-actin and anti-Bax antibody were purchased from Abcam (Shanghai, China). Goat anti-rabbit IgG (H+L), HRP-conjugated was purchased from Beijing TDY Biotech (Beijing, China).

### Cell Lines and Esophageal Cancer Animal Model

Three esophageal cancer cells lines (K510, K30, K150) were provided by Procell Life Science & Technology Co., Ltd. (Wuhan, China). All cells lines were cultured in 10% (w/w) FBS medium and incubated in incubator under 5% CO_2_ at 37°C. Male nude mice (4 weeks, approximately 16 g) were purchased from the Experimental Center of Xi'an Jiaotong University. Mice were acclimatized for 1 week under SPF conditions. Approximately 100 μL DMEM medium, containing 5 × 10^5^ K30 cells, was subcutaneously injected. The tumor-bearing mouse model was used in further experiments when the tumor grew to the appropriate volume. All animal experiments were carried out in compliance with the Guidelines for Use and Care of Animals at Xi'an Jiaotong University (Number XJTULAC 2016-412).

### Determination of Drugs Combination Optimal Synergy

Three esophageal cancer cells (K510, K30, and K150) were cultured in DMEM with 10% (w/w) FBS and incubated at 37°C with 5% CO_2_. The cells were treated with DOX and β-elemene at different dilution ratios (1:5; 1:10 and 1:15) for 72 h. Cell growth inhibition was measured by the CCK-8 method. During the plating, all samples had 3 duplicated wells. A 100 μL of leuco medium was added to each well, containing 10% (v/v) CCK-8. After incubation for 2 h, the absorbance for each well (at 450 mm) was obtained using a microplate reader. The drug concentration was determined by IC50, calculated using the GraphPad Prism 5 software. The combination index (CI) value reflects the combinable effect of the drugs. CalcuSyn program was used to calculate the CI according to the previous research (34). When the CI value was <0.9, the two drugs were considered to have a synergistic effect. When the CI value was >0.9 and <1.1, it was considered as a superposition effect, and when >1.1, it was considered as an antagonistic effect.

### Preparation and Characterizations of bMED NPs

β-elemene is adsorbed in the positively charged bMSN NPs to form bMSN@β-elemene and DOX forms a hybrid DOX@HA with the HA negatively charged surface. bMSN@β-elemene and DOX@HA are combined to form bMED NPs by electrostatic adsorption, which is negatively charged. The method for synthesizing bMED NPs was as follows: 9 mg of amino-modified bMSN NPs were dispersed in 5 mL of water, 1 mg β-elemene was slowly added and continuously stirred to prevent flocculent precipitation. React overnight. 0.1 mg of DOX was slowly added to the HA aqueous solution, and stirred for 10 min. Slowly added the synthesized bMSN@β-elemene to DOX@HA, and reacted for 2 h to obtain bMED NPs. All reactions were performed at room temperature. The size and potential of the bMED NPs were measured using a Malvern instrument and their morphology was measured by a transmission electron microscope (TEM). The encapsulation efficiency (EE) of β-elemene in bMED NPs was determined by high performance liquid chromatography and the drug loading (DL) of DOX was determined by fluorescence spectrophotometry. These values were calculated using the following formulas:

$$\begin{array}{l} \text{E}ncapsulation\;efficienc\text{y}=\frac{{\text{C}}_{\text{d}rug\;remai\text{n}}}{{\text{C}}_{\text{d}rug\;inpu\text{t}}}\times 100\%\;\\ \text{D}rug\;loading=\frac{{\text{C}}_{\text{d}rug\;remai\text{n}}}{{\text{C}}_{\text{b}MED\;NPs\;inpu\text{t}}}\times 100\%\; \end{array}$$

### *In vitro* Drug Release of bMED NPs

DOX drug release by bMED NPs was studied using dialysis. β-elemene is insoluble in water and therefore cannot be detected in the experiment to determine whether bMED NPs released it. Thus, DOX is mainly used for this test as it is water-soluble and fluorescent. The same concentration of bMED NPs, with 10 U mL^−1^ HAase and free DOX, was dialyzed as a control and with same conditions.

### *In vitro* Stability of bMED NPs

bMED NPs were dissolved in PBS (4 and 37°C), medium (37°C) and serum (37°C), to determine whether they were stable. This property was achieved by detecting the size of bMED NPs using a Malvern particle size analyzer.

### *In vitro* Cytotoxicity of bMED NPs

K510, K30, and K150 cells (1 × 10^4^) were seeded into 96-well sterile flat-bottomed culture plates (100 μL per well) and incubated for 24 h under a 5% CO_2_ atmosphere at 37°C. Then, bMSN NPs, bMED NPs and dual drugs solution was added to each well (DOX: 15 μg mL^−1^; β-elemene: 150 μg mL^−1^). The plates were incubated in an incubator for 48 h at 37°C with 5% CO_2_. Ten microliters of CCK-8 reagent was added to each well, and the plates were incubated for further 3 h. The optical density (OD) was measured at 450 nm using an ELISA plate reader (Infinite® 200 Pro, Tecan, Switzerland). The cell viability was calculated by the following formula:

$$\begin{array}{l} \text{C}\text{e}l\text{l}\;viability=\frac{{\text{O}\text{D}}_{\text{b}MED\;NP\text{s}}-{\text{O}\text{D}}_{\text{b}lan\text{k}}}{{\text{O}\text{D}}_{\text{c}ontro\text{l}}-{\text{O}\text{D}}_{\text{b}lan\text{k}}}\times 100\%\; \end{array}$$

### Colony Formation and Transwell Migration Assays

Colony formation assay: the ability of K30 cells to proliferate, under different drug treatments, was examined by the cell colony formation assay. The cells were uniformly dispersed, equal numbered and cultured into 5 culture dishes. After incubation at 37°C with 5% CO_2_ for 24 h, the medium was replaced and a new one was added with DOX, β-elemene, dual drug and bMED NPs (C_DOX_: 30 μg mL^−1^ and C_β−elemene_: 300 μg mL^−1^). DMSO was used as a control. The cell cultures were terminated when visible clones appeared in the culture dishes. The supernatants were discarded, and the cells carefully washed twice with PBS and fixed in 4% paraformaldehyde for 15 min. After fixative removal, the cells were stained with an appropriate amount of crystal violet for 10–30 min. Finally, the staining solution was washed away with PBS and photographed to calculate the number of clones using Image J.

Transwell migration assay: the amount of K30 migrating cells was used to evaluate the anti-migration ability of the drugs. The cells were treated with DOX, β-elemene, dual drugs, and bMED NPs (C_DOX_: 6 μg mL^−1^ and C_β_−elemene: 60 μg mL^−1^) with DMSO treated cells used as a control. The cells were suspended in serum-free medium and their number was adjusted to 4 × 10^4^ cells. The medium with 10% serum was added in the lower chamber and at the bottom of the 24-well plate, and the cell suspension was added to the upper chamber. After 24 h incubation, the chamber was removed with forceps, the upper chamber fluid blotted, and transferred to a well containing 800 μL of Giemsa stain and incubated for 10–30 min at room temperature. Five visually selected fields were randomly determined, under the microscope, for statistical analysis.

### Cell Uptake of bMED NPs

In order to detect the cell uptake behavior of bMED NPs, coumarin 6 replaced β-elemene in the pore of bMSN NPs (bMCD NPs) and HA was used as blocker. The samples were added and incubated with the cells for 48 h. The cells were fixed for 10 min with paraformaldehyde (4%) and the nuclei labeled with DAPI. The cell internalization of bMCD NPs was observed by confocal microscopy (TCS SP5 II, Leica, Germany).

### Western Blot

After treating K30 cells with saline, DOX, β-elemene, dual drugs, bMED NPs for 48 h, the cells were collected by centrifugation and lysed with radio immunoprecipitation assay (RIPA, Beyotime) lysis buffer supplemented with 1% phenylmethylsulfonyl fluoride (PMSF). The proteins were separated by SDS-PAGE gel and semi-dry transferred to a nitrocellulose membrane. After washing the membrane with TBS-Tween-20 (TBST), to block proteins non-specific, the membrane was soaked in a 5% skimmed milk blocking solution at 4°C overnight. The membrane was washed and incubated with the primary antibody (in blocking solution) for 1 h with shaking. This step was followed by membrane washing and incubation with the secondary antibody was incubated (on a shaker) for 1 h. Finally, the reaction band was observed with an enhanced chemiluminescence reagent (Pierce).

### Flow Cytometry Analysis

Apoptosis was detected using flow cytometry. The quantitative detection of phosphatidylserine on the surface of apoptotic cells was performed using Annexin-V-FITC and PI staining. The cells were incubated with DOX, β*-*elemene, dual drugs and bMED NPs for 48 h and the cells were treated with saline as control (C_DOX_: 3 μg mL^−1^ and C_β−elemene_: 30 μg mL^−1^), and the cells were harvested by centrifugation at 800 rpm for 5 min, washed with PBS, centrifuged again and resuspended in PBS. The cells were stained with Annexin-V-FITC for 1 h, stained with PI for 30 min and analyzed by a FACScan system.

### Hemolysis Test

Mice whole blood were taken and the blood samples were divided into triton X-100 (1% v/v), bMSN NPs, bMED NPs, dual drug and DMSO (0.5% v/v) groups. After incubating for 2 h at 37°C, the blood samples were centrifuged at 13,000 rpm for 15 min and the supernatants were dissolved and measured by a UV-Vis spectrophotometer (UV 2900, Shanghai) at a wavelength of 394 nm. All animal experiments were approved by the Experimental Animal Management Committee of Xi'an Medical University.

### *In vivo* Toxicity of bMED NPs

20 mice (10 males and 10 females) were randomly divided into two groups. After 3 days of feeding under SPF conditions, bMED NPs and dual drugs were intravenously injected (contained drug concentration: DOX: 15 mg kg^−1^, β-elemene: 150 mg kg^−1^). Two days after the administration, mice vital signs were observed. Mice weights and deaths were continuously recorded, and mortality was calculated within 14 days. Heart, liver and kidney tissues were collected for histopathology analysis.

### *In vivo* Distribution of bMED NPs

The animal models were injected in the tail vein with IR780-loaded bMSN NPs (10 mg mL^−1^, 200 μL) and with the same amount of free IR780 for the control group. IR780 was adsorbed in the mesopores of bMSN NPs, which was achieved by hydrophobic adsorption, according to the synthesis method of bMSN@β-elemene. DOX was still adsorbed on the surface of bMSN NPs, through the electrostatic adsorption. The fluorescence distribution in mice was observed at different time points using the IVIS Imaging System with the excitation wavelength is 780 nm and the emission wavelength is 845 nm. At the end of imaging, all the mice were euthanized and the hearts, livers, spleens, lungs, kidneys and tumors were collected. The fluorescent signals in the tissues were detected under the same conditions.

### *In vivo* Antitumor Effects of bMED NPs

The *in vivo* tumor inhibition effects of bMED NPs were studied using 30 tumor-bearing mice. Mice were randomly divided into 5 groups of 6 animals each. Mice treatments started when the tumor volume reached the appropriate level. Each group of mice was intravenously injected with the same dose of saline, β*-*elemene, DOX, dual drugs and bMED NPs twice a week for 3 weeks. During this period, body weight and tumors size were continuously recorded. After 3 weeks, removed all the tumor tissues from mice in each group and measured the tumor size and weight. The tumor tissues were embedded in paraffin and cut into 5 μm thick slices. Hematoxylin-eosin (H&E) staining and TUNEL staining were used to detect tumor cell morphology and apoptosis. TUNEL staining was conducted with a TUNEL apoptosis assay kit (Beyotime Biotechnology, Shanghai, China), and the experiment was performed according to the instructions. The paraffin sections were dewaxed and then 20 μg mL^−1^ of proteinase k was added and took effect at 37°C for 15 min. The excess proteinase k was washed away with PBS, and the sections were incubated in 3% hydrogen peroxide at room temperature for 20 min, and washed with PBS for three times. Fluorescence microscopy was used to analyze the results.

### Statistical Analysis

Data are expressed as the mean ± SD of three independent replicates, which was calculated with the Graphpad Prism 5.0 software. When the *P*-value <0.05, it was considered to represent a statistically significant difference between comparative data. And the statistical differences between the means were analyzed by the Student's *t*-test. The significance was set to 5%.

## Results

### Synergistic Effect of DOX and β-Elemene

The combination of DOX and β-elemene were incubated with the cells (K 510, K30, and K 150) at the proportion of 1:5, 1:10, and 1:15, and the cytotoxicity of all three cell lines was increased. The CI was calculated by the CompuSyn software. As shown in Table 1, the CI values of the three treated cell lines were <0.9, indicating that DOX and β-elemene have excellent synergistic effects. When DOX with β-elemene were mixed in proportion of 1:10, the CI value was the smallest, indicating that this concentration has the greatest cytotoxicity for the three cell lines. Therefore, when DOX and β-elemene were combined at a ratio of 1:10, the optimal synergistic ratio was achieved. The highest sensitivity was observed in the K30 cell line. The drugs CI values and concentrations for K510, K30, and K150 inhibition using the combinations DOX and β-elemene at the proportions of 1:5 and 1:15, are shown in Tables S1, S2.

**Table 1:** CI and dose reduction values for inhibition on K510, K30, and K150 by combining doxorubicin with β-elemene in proportion of 1:10.

**% Inhibition**	**CI**	**Doxorubicin**			**β-elemene**		
		**Conc.:(μM)**		**Dose reduction**	**Conc.:(μM)**		**Dose reduction**
		**Alone**	**Mix**		**Alone**	**Mix**	
K510							
50	0.6463	27.87	5.881	4.739	355.5	57.32	6.202
75	0.7332	49.43	9.537	5.183	835.1	94.77	8.812
95	0.7643	99.87	18.53	5.390	1334	179.2	7.444
K30							
50	0.6354	38.14	9.222	4.136	478.9	93.24	5.136
75	0.6112	71.31	20.36	3.502	992.7	200.7	4.946
95	0.5895	110.3	32.63	3.380	1413	331.4	4.264
K150							
50	0.6432	31.46	7.321	4.297	297.4	77.32	3.846
75	0.6752	65.82	15.24	4.319	584.2	154.4	3.784
95	0.7743	104.5	20.57	5.080	1067	201.6	5.293

### Preparation and Characterization of bMED NPs

**Figure 1:**
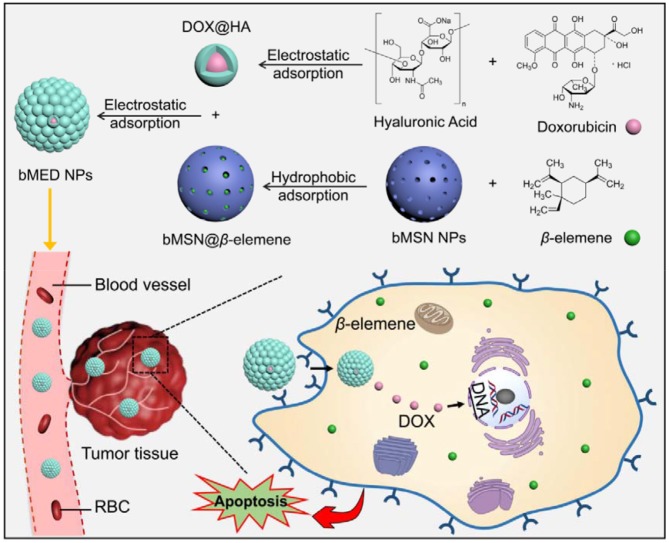
Synthesis of bMED NPs.

**Figure 2:**
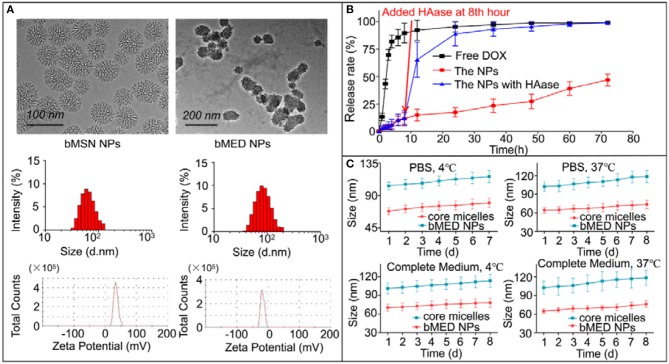
Characterization of bMED NPs: **(A)** Morphology, size and zeta potential of bMSN NPs and bMED NPs; **(B)** *In vitro* DOX releasing of free DOX, bMED NPs, and bMED NPs with HAase; **(C)** *In vitro* stability of bMED NPs in different temperature and different solvent. The data reported are the mean ± SD for triplicate samples.

The bMED NPs were prepared according to the protocol described in Figure 1. First, DOX@HA and bMSN@β*-*elemene were synthesized. Next, we assembled bMED NPs by electrostatic adsorption, in which β*-*elemene in the pore of bMSN NPs and DOX@HA was adsorbed around bMSN@β*-*elemene. As shown in Figure 2A, the shapes of bMSN NPs and bMED NPs were spherical and their particle sizes were 56.05 ± 5.78 and 77.15 ± 8.61 nm, respectively. And average hydrodynamic diameter were 59.19 ± 8.66 and 91.94 ± 13.46 nm, respectively. The nanocarriers showed uniform particle size distributions, as expected. The change of zeta potential proved to be a packaging process. The results showed that the zeta potentials of bMSN NPs and bMED NPs are 34.2 ± 1.06 and−17.1 ± 0.5 mV.

The bMED NPs EE and DL were measured and calculated using high performance liquid chromatography (HPLC). The maximum EE and DL of β-elemene were 96.7 ± 2.7% and 9.9 ± 1.4%; while, the EE and DL of DOX were 85.2 ± 6.2% and 1.1 ± 0.3%. Figure 2B showed the release curve of DOX in bMED NPs. The bMED NPs with HAase and free DOX were used as control. As shown, the free DOX group has a burst release with a rate of up to 92% at 12 h. The DOX release in the bMED NPs group was slower with only 46.89% of the DOX released at 72 h. Significantly, HAase could increase the ability of bMED NPs to release DOX. When HAase was added, DOX in bMED NPs was rapidly released, and at 24 h, the release rate increased to 88.79%. As shown in Figure 2C, the stability studies were performed by measuring changes in the hydrodynamic diameters of bMED NPs at different times. The bMED NPs remained stable in PBS at 4 and 37°C, and the size did not change. The nanoparticles also remained stable in complete medium and FBS at 37 °C.

### Colony Formation and Transwell Migration Assays

**Figure 3:**
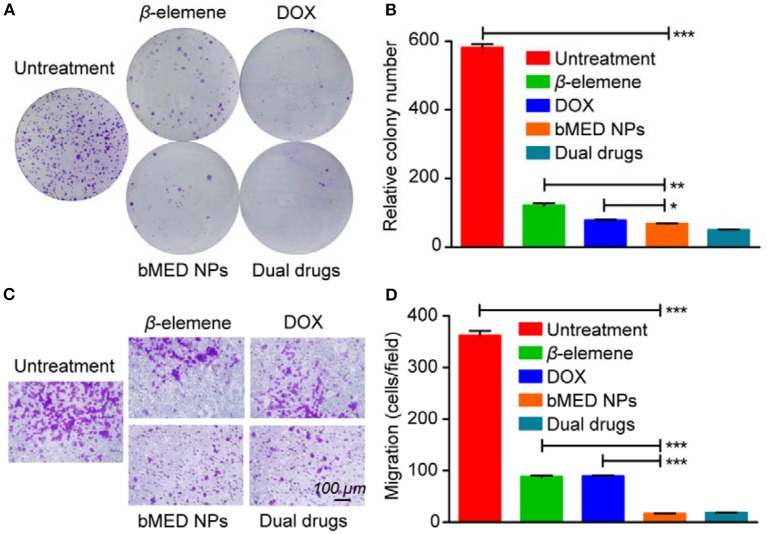
The anti-proliferation and anti-migration effect assay in K30 cells when treated with β-elemene, DOX, dual drugs and bMED NPs: **(A)** Clony images of cells; **(B)** Quantitative data on the relative colony number; **(C)** The images of cells in transwell migration assay; **(D)** Quantitative data on transwell migration. Error bars represent the SD of the mean. *p < 0.05, **p < 0.01, ***p < 0.001.

We obtained images of the colony formation (Figure 3A) and counted the number of colonies (Figure 3B). As shown, the control cells had the highest colonies number. The β*-*elemene and DOX groups were relatively reduced, with fewer in dual drug groups and minimal bMED NPs groups. Moreover, the drug treated group was significantly different from the DMSO treated group. When treated with bMED NPs, the proliferative capacity of K30 cells was almost completely inhibited. As shown in Figures 3C,D, the migration ability of K30 cells, after treatment with the different samples, was significantly attenuated. The migration ability of cells became very low when treated with bMED NPs.

### *In vitro* Cytotoxicity and Cellular Uptake of bMED NPs

**Figure 4:**
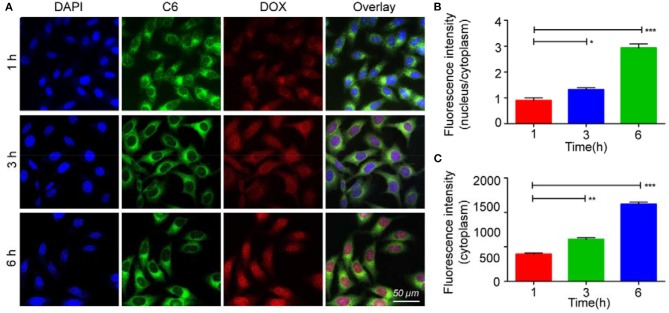
*In vitro* internalization of bMCD NPs: **(A)** The K30 cells were incubated with dual-fluorescence bMCD NPs for 1 h, 3 h and 6 h; **(B)** The ratio of the fluorescence intensity of the nucleus to the cytoplasm represents that DOX is released and enters the nucleus from the cytoplasm; **(C)** Changes in the fluorescence intensity of coumarin 6 in the cytoplasm over time. Error bars represent the SD of the mean. *p < 0.05, **p < 0.01, ***p < 0.001.

**Figure 5:**
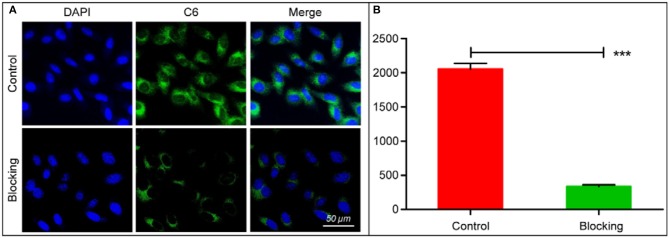
*In vitro* cell affinity of bMCD NPs: **(A)** Fluorescent images of cells before and after blocking with HA. Cell nucleus stained with DAPI. **(B)** Histogram showed cell fluorescence integrated density of bMCD NPs and bMCD NPs with HA blocking. The data reported are the mean ± SD for triplicate samples; ***p < 0.001.

The cytotoxicity of bMED NPs for K510, K30, and K150 cells was detected by the CCK-8 method. As shown in Figure S1, bMSN NPs has no obvious cytotoxicity and there was no significant difference in cytotoxicity between bMED NPs and dual drugs. In the cellular uptake experiment, coumarin 6 was used to replace β*-*elemene, and was loaded in the pore of bMSN NPs to form bMCD NPs with double fluorescence (DOX has red fluorescence). Figure 4 showed bMCD NPs internalization in the K30 cells. As shown in Figure 4A, the dynamic distribution of coumarin 6 and DOX, during the internalization process, was significantly different. In the first hour, the DOX and coumarin 6 mainly distributed in the cytoplasm. In the whole internalization process, the fluorescence of coumarin 6 was basically distributed in the cytoplasm and the fluorescence intensity gradually increased. However, the fluorescence signal distribution of DOX changed after 3 and 6 h. After 3 h, the DOX signal gradually spread from the cytoplasm to the whole cell. Six hours later, the fluorescence of DOX accumulated in the nucleus. Figure 4B showed the nucleus/cytoplasm fluorescence intensity ratio of DOX. With time, the ratio gradually increased, indicating that the DOX signal gradually accumulated and strengthened into the nucleus. Figure 4C showed a quantitative analysis of the fluorescence intensity of the coumarin 6 in the cytoplasm, which corresponded to the result of the confocal images shown in Figure 4A. Figure 5 showed an overall picture of cell uptake, with or without HA blocking. During the experiment, 1% HA was added to the medium before incubation with bMCD NPs and after 1 h of incubation, a distinct fluorescence was observed in the cytoplasm. On the contrary, only weak fluorescence was observed in the blocking group.

### Western Blot and Flow Cytometry Analysis

As shown in Figure S2A, K30 cells that were treated with the bMED NPs had an upregulation of Bax protein expression and a downregulation of Bcl-2 protein expression. Figures S2B–D showed quantitative analyses of Bax and Bcl-2 proteins expression, with the gradual decrease of Bcl-2 protein expression and increase of Bax protein expression in the bMED NPs groups. The results of flow cytometry (Figure S2E) showed a greater apoptosis in the dual drugs group compared to DOX and β-elemene groups. In the bMED NPs group, apoptosis was also greater than that in the dual drugs group.

### *In vivo* Toxicity of bMED NPs

**Figure 6:**
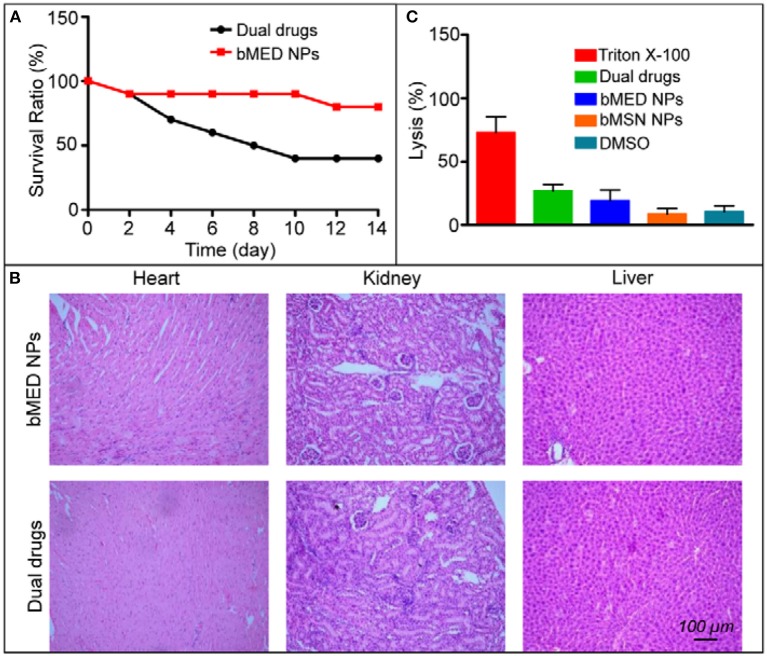
*In vivo* toxicity results of bMED NPs: **(A)** Survival rate of free dual drugs and bMED NPs; **(B)** The H&E staining results of pathological examination of the heart, kidney and liver treated with dual drugs and bMED NPs; **(C)** The results of hemolysis analysis.

A major advantage of anti-tumor nanocarriers is their ability to reduce non-specific toxicity for normal organs and tissues. Figure 6A showed the acute toxicity results of bMED NPs and dual drugs. After 14 days, the survival rate of mice was 80% in the bMED NPs treatment group; while, the survival rate was only 40% in the dual drugs group. The acute toxicity of bMED NPs was significantly lower than that with dual drugs. Fourteen days later, the mice were euthanized and the hearts, kidneys and livers collected, sectioned and stained. As shown in Figure 6B, the organs of mice that were treated with bMED NPs had less damage compared to the dual drugs group. The result of the hemolysis test showed that bMED NPs had lower hemolytic toxicity relative to triton-X and the dual drugs treatment group (Figure 6C).

### *In vivo* Distribution and Antitumor Effects of bMED NPs

**Figure 7:**
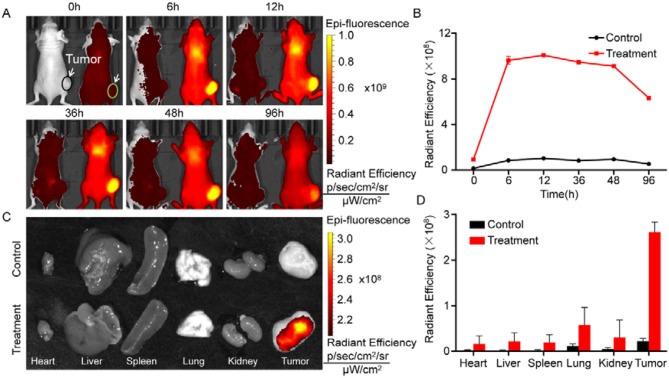
*In vivo* distribution of bMED NPs. **(A)** Fluorescent images of mice within 96 hours. The right mouse was injected IR780-loaded bMSN NPs, meanwhile, the left mouse was injected free IR780 dye. The arrows were pointed to tumors; **(B)** Line graph of fluorescence intensity at tumor sites; **(C)** Fluorescent images of major organs and tumor tissues; **(D)** Quantification of fluorescence intensity in major organs and tumor tissues.

IR780 was attached to the surface of the bMED NPs. The esophageal cancer animal model was used to detect the *in vivo* distribution of bMED NPs. Figure 7A showed fluorescence images of mice at different time points after their injection with same amounts of IR780-loaded bMED NPs and free IR780. The arrow pointed to the tumors. At 6 h, the fluorescence was accumulated in the livers and tumors of the IR780-loaded bMED NPs mice group, and the fluorescence in the tumors was much stronger than in the livers. At 12 h, the fluorescence continued to accumulate in the tumor sites, and the fluorescence intensity of the livers gradually increased. The strongest fluorescence was at 12 h. In the free-IR780 group, no significant signal accumulation was observed in the tumors. Figure 7B showed that the bMED NPs significantly prolonged the *in vivo* circulation time of IR780. At the end of the imaging period, all mice were sacrificed and the hearts, livers, spleens, lungs, kidneys and tumors were taken. The fluorescence in the tissues was detected under the same conditions. As shown in Figure 7C, the fluorescence mainly accumulated in the tumors and there were few fluorescence signals in other organs, which could be ignored. Figure 7D showed the fluorescence intensity of the major organs, which is consistent with the *in vivo* imaging results.

**Figure 8:**
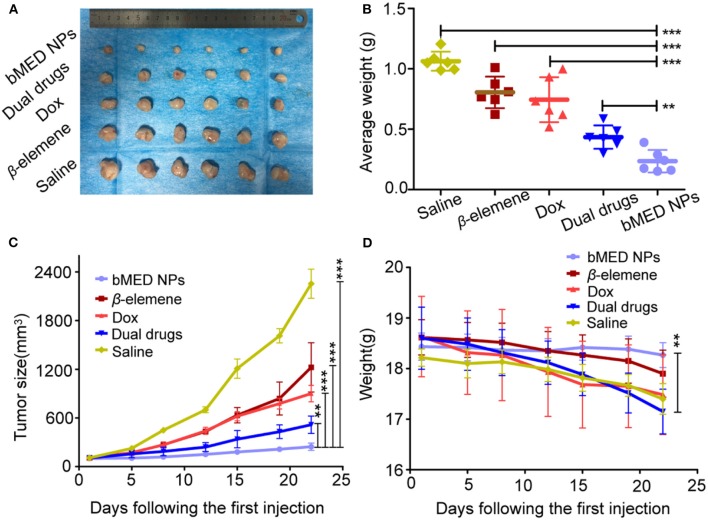
*In vivo* tumor inhibition of bMED NPs: **(A)** The tumors images in different groups; **(B)** The tumor weight curves of mice in each group; **(C)** Tumor size curves in each treatment groups; **(D)** The body weight curves of mice in each group. Error bars represent the SD of the mean. **p < 0.01, ***p < 0.001.

The tumor volume is intuitively a response to the *in vivo* antitumor effects. Therefore, tumors size and body weight of tumor-bearing mice were measured after injection. The tumor growth curve was shown in Figure 8C. The tumors were the smallest in the bMED NPs group. The tumor size of the dual drugs group was smaller than those in the single drug group. The mean tumor volume was the largest in the saline group. As shown in Figure 8D, the body weight of mice in the DOX treatment group was less than that in the β-elemene treatment group, due to DOX high toxicity; while, β-elemene was less toxic. There was no significant decrease in body weight and saline in the bMED NPs group, indicating that bMED NPs can reduce drug toxicity. Figure 8B showed the weight of the tumors corresponding to Figure 8A. Statistically, bMED NPs group was significantly different from saline group (^***^p < 0.001), the bMED NPs group showed a significant anti-tumor effect than in the dual drugs group and the dual drugs group was more effective than the DOX and β-elemene groups.

### Pathological Section

To test the potential *in vivo* toxicity of bMED NPs, the mice were sacrificed and tumor tissues were taken and stained with H&E for histological analysis. As shown in Figure S3A, there was no significant damage in the saline group. The tissue necrotic area of the β-elemene, DOX and dual drugs groups increased. The organs in the bMED NPs treatment group were healthier than those in the dual drugs group. TUNEL staining was used to detect apoptosis and the results showed that treatment with bMED NPs could significantly induce apoptosis and inhibit tumor growth (Figure S3B). Quantification of TUNEL staining also showed the consistent results (Figure S4).

## Discussion

Esophageal cancer is one of the four major cancers in China that has high incidence and low five-year survival rate (2). Combination therapy is a very important tool in cancer treatment (35–37). Our previous studies have shown that β-elemene and DOX have a combination therapeutic effect. However, due to the difference in pharmacokinetics between the β-elemene and DOX, it is difficult to determine the optimal dose and drug ratio to exert optimal therapeutic effects at the tumor site. The combination of multiple drugs may produce better antitumor effects and the assessment of drug-drug interactions are critical (38). CI analysis is a common method for assessing the drugs interactions in combination therapy. The synergistic effect of DOX and β-elemene, as a dual drug delivery system, was verified by measuring CI. The CI value was <0.9 when the ratio of DOX to β-elemene was 1:10, which indicated that DOX and β-elemene had good synergistic effect and can be used as drugs of choice for combination therapy of esophageal cancer.

The bMSN NPs have larger pores size and specific surface areas, which make them good carriers for combination therapy (39). They can also be loaded with DOX and β-elemene in a determined ratio. Among bMSN NPs, the HA shell encapsulates DOX by electrostatic adsorption (DOX@HA). β-elemene is loaded into bMSN NPs pores by hydrophobic interaction. DOX@HA is then loaded onto the surface of the bMSN NPs by electrostatic adsorption. The synthesized bMED NPs have a particle size of 77 nm, which guaranteed an improved accumulation at the tumor sites through the EPR effect. The negative potential of bMED NPs also provides a better stability in the circulatory system *in vivo*. The drug loading of the nanocarriers is very important for a targeted delivery of the drug. The larger pore size and superficial area of the bMSN NPs allow the maximization of DOX and β-elemene loading, which leads to an efficient drug delivery *in vivo*, their larger particle size can also extend the time of *in vivo* drug delivery. To assess the drug delivery capacity of bMED NPs at the cellular level, cell uptake and affinity experiments were performed. To observe the cellular uptake of bMED NPs, the hydrophobic green fluorescent dye coumarin 6 was encapsulated into the pore of bMSN NPs. Coumarin 6 has a strong fluorescence and affinity to cell membranes (40) and can be used for dual fluorescence under a confocal microscope when used with DOX red fluorescence. The dual fluorescence signal of the coumarin 6 and DOX-loaded bMSN nanoparticles (bMCD NPs) can show cell uptake of the dual drug nanoparticles. Over time (0–6 h), bMCD NPs entered the cytoplasm by endocytosis, which was consistent with previous research results (41). As bMCD NPs decomposed, DOX was released and gradually entered into the nucleus. Since DOX binds mainly to the nuclear DNA, it could be observed that the fluorescent signal gradually shifts from the cytoplasm to the nucleus. After bMCD NPs decomposition, the coumarin 6 in the pore of bMSN NPs is gradually released, through the cell membrane and into the cytoplasm. Therefore, a gradual increase in the green fluorescence of coumarin 6 can be observed. These results indicated that bMED NPs could achieve efficient delivery of β-elemene and DOX based on their synergistic effect. The results of the affinity experiment showed that the green fluorescence of coumarin 6 was very weak compared with the control group after HA addition. The group without HA showed a strong fluorescent signal 1 h after bMED NPs treatment. These results indicated that bMED NPs had a good ability to target esophageal cancer cells and indicated that its targeting was caused by HA. This can be explained by HA function as CD44 protein receptor, which is highly expressed in various cancers, including esophageal cancer (42, 43). In addition, the release efficiency of DOX in bMED NPs has also been studied to assess the drug delivery capacity of bMED NPs. The addition of HAase mimics the *in vivo* environment and significantly increase the efficiency of DOX release; thereby, demonstrating that the presence of HA provides bMED NPs with good targeting and drug delivery capabilities. To verify the distribution and targeting ability of bMED NPs *in vivo*, the changes in fluorescent signals, provided by surface modified IR780, were monitored in a subcutaneous tumor model of esophageal cancer for 96 h. The results showed that bMED NPs were well-targeted to tumors and effectively prolonged the circulating time *in vivo* compared to small molecule drugs alone, which was demonstrated from the efficiency of bMSN nanocarriers. Up to 96 h, the tumor site showed a strong fluorescence intensity, demonstrating targeting by bMED NPs and an *in vivo* long-term circulation, which were also due to the effects of HA and the stability of bMED NPs. The results *in vivo* were consistent with those *in vitro*.

To further verify the antitumor effects of bMED NPs, three esophageal cancer cell lines were selected (K510, K30, and K150) and the cytotoxicity of bMED NPs was detected by CCK-8 analysis. For the three cell lines, there was almost no cytotoxicity detected; however, approximatively 50% of the cells died after administration of the dual drug and bMED NPs, suggesting that the cytotoxicity is due to the cytotoxicity of the dual drugs. To assess bMED NPs antitumor effects, it was necessary to detect the *in vivo* toxicity of bMED NPs. The results of *in vivo* acute toxicity did not show significant toxicity, demonstrating that bMSN nanocarriers were effective in reducing the non-specific toxicity of DOX and β-elemene to normal tissues and organs. Tissue sections staining also showed no significant tissue damage compared to the dual drugs treatment group. These results further indicated that bMED NPs could be used for tumors combined treatment.

To study the *in vivo* antitumor effects of bMED NPs, changes in mice body weight were monitored within 22 days, and the results showed that the mice in the bMED treatment group had substantially no change in body weight compared with the dual drugs treatment and the single drug treatment groups. This may be due to the cardiotoxicity of DOX and the liver metabolism of the hydrophobic β-elemene. In addition, from the tumor size of each group of mice dissected after 22 days, the tumors of the bMED treatment group were the smallest and the therapeutic effects were the best. The dual drug treatment group also showed good antitumor effects by inhibiting tumor growth. However, due to the rapid metabolism rate of small molecule drugs, it was difficult for the dual drugs therapy to continue exerting antitumor effects; thereby, a weaker tumor suppressing effect was observed compared with the bMED NPs treatment group. Tumor weighing and volume calculation also showed similar results. The *in vivo* efficient antitumor effect was also attributed to the targeted drug delivery of bMED NPs, whose sustained and effective drug delivery may help deliver the drugs to the tumor site for better antitumor effects. These results indicated that bMED NPs had good antitumor effects *in vivo*. The results of the studies on the antitumor mechanism of bMED NPs showed that bMED can downregulate the expression of Bcl-2, increase the ratio of Bcl-2/Bax and cause apoptosis. This is consistent with the results of previous studies. H&E and TUNEL staining of tumor tissues also showed that bMED NPs had the best therapeutic effects. The above results indicated that bMED NPs had good antitumor effects *in vitro* and *in vivo* and could provide a good nanodrug platform for the treatment of esophageal cancer.

## Data Availability Statement

The raw data supporting the conclusions of this article will be made available by the authors, without undue reservation, to any qualified researcher.

## Ethics Statement

The animal study was reviewed and approved by Xian Jiaotong University Animal Care and Use Committee.

## Author Contributions

WZ and HL designed the experiments. WZ, HL, and YG performed the experiments. YG and GD analyzed the data. HL and YG wrote the manuscript. YW and DZ provided guidance of the project. All authors have read and agreed to the published version of the manuscript.

## Conflict of Interest

The authors declare that the research was conducted in the absence of any commercial or financial relationships that could be construed as a potential conflict of interest.

## References

[1] Arnal MJD Arenas AF Arbeloa AL Esophageal cancer: risk factors, screening and endoscopic treatment in Western and Eastern countries. World J Gastroentero. (2015) 21:7933–43. 10.3748/wjg.v21.i26.7933 PMC4499337 26185366

[2] Bray F Ferlay J Soerjomataram I Siegel RL Torre LA Jemal A. Global cancer statistics 2018: GLOBOCAN estimates of incidence and mortality worldwide for 36 cancers in 185 countries. Ca-Cancer J Clin. (2018) 68:394–424. 10.3322/caac.21492 30207593

[3] Kano M Seki N Kikkawa N Fujimura L Hoshino I Akutsu Y. miR-145, miR-133a and miR-133b: tumor-suppressive miRNAs target FSCN1 in esophageal squamous cell carcinoma. Int J Cancer. (2010) 127:2804–14. 10.1002/ijc.25284 21351259

[4] Lin JL. T1 esophageal cancer, request an endoscopic mucosal resection (EMR) for in-depth review. J Thorac Dis. (2013) 5:353–6. 10.3978/j.issn.2072-1439.2013.06.03 23825773 PMC3698294

[5] Gockel I Sgourakis G Lyros O Polotzek U Schimanski CC Lang H. Risk of lymph node metastasis in submucosal esophageal cancer: a review of surgically resected patients. Expert Rev Gastroent. (2011) 5:371–84. 10.1586/egh.11.33 21651355

[6] Mohri J Katada C Ueda M Sugawara M Yamashita K Moriya H. Predisposing factors for chemotherapy-induced nephrotoxicity in patients with advanced esophageal cancer who received combination chemotherapy with docetaxel, cisplatin, and 5-fluorouracil. J Trans Int Med. (2018) 6:32–7. 10.2478/jtim-2018-0007 29607302 PMC5874485

[7] Larkin J Chiarion-Sileni V Gonzalez R Grob JJ Cowey CL Lao CD Combined nivolumab and ipilimumab or monotherapy in untreated melanoma. New Engl J Med. (2015) 373:23–34. 10.1056/NEJMoa1504030 26027431 PMC5698905

[8] Douillard JY Cunningham D Roth AD Navarro M James RD Karasek P. Irinotecan combined with fluorouracil compared with fluorouracil alone as first-line treatment for metastatic colorectal cancer: a multicentre randomised trial. Lancet. (2000) 355:1041–7. 10.1016/S0140-6736(00)02034-1 10744089

[9] Greco F Vicent MJ. Combination therapy: opportunities and challenges for polymer-drug conjugates as anticancer nanomedicines. Adv Drug Deliver Rev. (2009) 61:1203–13. 10.1016/j.addr.2009.05.006 19699247

[10] Enzinger PC Burtness BA Niedzwiecki D Ye X Douglas K Ilson DH. CALGB 80403 (Alliance)/E1206: a randomized phase II study of three chemotherapy regimens plus cetuximab in metastatic esophageal and gastroesophageal junction cancers. J Clin Oncol. (2016) 34:2736–42. 10.1200/JCO.2015.65.5092 27382098 PMC5019745

[11] Suntharalingam M Winter K Ilson D Dicker AP Kachnic L Konski A. Effect of the addition of cetuximab to paclitaxel, cisplatin, and radiation therapy for patients with esophageal cancer the NRG oncology RTOG 0436 Phase 3 randomized clinical trial. JAMA Oncol. (2017) 3:1520–8. 10.1001/jamaoncol.2017.1598 28687830 PMC5710193

[12] Gewirtz DA. A critical evaluation of the mechanisms of action proposed for the antitumor effects of the anthracycline antibiotics adriamycin and daunorubicin. Biochem Pharmacol. (1999) 57:727–41. 10.1016/S0006-2952(98)00307-4 10075079

[13] Xiong XB Ma ZS Lai R Lavasanifar A. The therapeutic response to multifunctional polymeric nano-conjugates in the targeted cellular and subcellular delivery of doxorubicin. Biomaterials. (2010) 31:757–68. 10.1016/j.biomaterials.2009.09.080 19818492

[14] Ross P Nicolson M Cunningham D Valle J Seymour M Harper P Prospective randomized trial comparing mitomycin, cisplatin, and protracted venous-infusion fluorouracil (PV1 5-FU) with epirubicin, cisplatin, and PV15-FU in advanced esophagogastric cancer. J Clin Oncol. (2002) 20:1996–2004. 10.1200/JCO.2002.08.105 11956258

[15] Zhang L Yao MC Yan W Liu XN Jiang BF Qian ZY. Delivery of a chemotherapeutic drug using novel hollow carbon spheres for esophageal cancer treatment. Int J Nanomed. (2017) 12:6759–69. 10.2147/IJN.S142916 28932119 PMC5600264

[16] Singal PK Iliskovic N. Doxorubicin-induced cardiomyopathy. New Engl J Med. (1998) 339:900–5. 10.1056/NEJM199809243391307 9744975

[17] Honda M Miura A Izumi Y Kato T Ryotokuji T Monma K. Doxorubicin, cisplatin, and fluorouracil combination therapy for metastatic esophageal squamous cell carcinoma. Dis Esophagus. (2010) 23:641–5. 10.1111/j.1442-2050.2010.01070.x 20545978

[18] Lee HH Ye S Li XJ Lee KB Park MH Kim SM. Combination treatment with paclitaxel and doxorubicin inhibits growth of human esophageal squamous cancer cells by inactivation of Akt. Oncol Rep. (2014) 31:183–8. 10.3892/or.2013.2851 24247637

[19] Chen WX Lu Y Wu JM Gao M Wang AY Xu B. β-elemene inhibits melanoma growth and metastasis via suppressing vascular endothelial growth factor-mediated angiogenesis. Cancer Chemoth Pharm. (2011) 67:799–808. 10.1007/s00280-010-1378-x 20563582

[20] Li QQ Wang GD Huang FR Banda M Reed E. Antineoplastic effect of beta-elemene on prostate cancer cells and other types of solid tumour cells. J Pharm Pharmacol. (2010) 62:1018–27. 10.1111/j.2042-7158.2010.01135.x 20663036

[21] Liu J Zhang Y Qu JL Xu L Hou KZ Zhang JD. β-Elemene-induced autophagy protects human gastric cancer cells from undergoing apoptosis. Bmc Cancer. (2011) 11:1–10. 10.1186/1471-2407-11-183 21595977 PMC3115914

[22] Zhan YH Liu J Qu XJ Hou KZ Wang KF Liu YP. β-elemene induces apoptosis in human renal-cell carcinoma 786-0 cells through inhibition of MAPK/ERK and PI3K/Akt/mTOR signalling pathways. Asian Pac J Cancer P. (2012) 13:2739–44. 10.7314/APJCP.2012.13.6.2739 22938451

[23] Chang ZW Gao M Zhang WJ Song LJ Jia YX Qin YR. β-elemene treatment is associated with improved outcomes of patients with esophageal squamous cell carcinoma. Surg Oncol. (2017) 26:333–7. 10.1016/j.suronc.2017.07.002 29113648

[24] Zhai BT Zeng YY Zeng ZW Zhang NN Li CX Zeng YJ. Drug delivery systems for elemene, its main active ingredient beta-elemene, and its derivatives in cancer therapy. Int J Nanomed. (2018) 13:6279–96. 10.2147/IJN.S174527 30349250 PMC6186893

[25] Cao C Wang Q Liu Y. Lung cancer combination therapy: doxorubicin and beta-elemene co-loaded, pH-sensitive nanostructured lipid carriers. Drug Des Dev Ther. (2019) 13:1087–98. 10.2147/DDDT.S198003 31118562 PMC6498957

[26] Agudelo D Bourassa P Berube G Tajmir-Riahi HA. Review on the binding of anticancer drug doxorubicin with DNA and tRNA: structural models and antitumor activity. J Photoch Photobio B. (2016) 158:274–9. 10.1016/j.jphotobiol.2016.02.032 26971631

[27] Li CL Chang L Guo L Zhao D Liu HB Wang QS. β-elemene induces caspase-dependent apoptosis in human glioma cells in vitro through the upregulation of bax and Fas/FasL and downregulation of Bcl-2. Asian Pac J Cancer P. (2014) 15:10407–12. 10.7314/APJCP.2014.15.23.10407 25556484

[28] Dai SJ Ye ZM Wang FZ Yan FQ Wang L Fang J. Doxorubicin-loaded poly(epsilon-caprolactone)-Pluronic micelle for targeted therapy of esophageal cancer. J Cell Biochem. (2018) 119:9017–27. 10.1002/jcb.27159 30256436

[29] Kolishetti N Dhar S Valencia PM Lin LQ Karnik R Lippard SJ. Engineering of self-assembled nanoparticle platform for precisely controlled combination drug therapy. Proc Natl Acad Sci USA. (2010) 107:17939–44. 10.1073/pnas.1011368107 20921363 PMC2964221

[30] Hu CMJ Zhang LF. Nanoparticle-based combination therapy toward overcoming drug resistance in cancer. Biochem Pharmacol. (2012) 83:1104–11. 10.1016/j.bcp.2012.01.008 22285912

[31] Estanqueiro M Amaral MH Conceicao J Lobo JMS. Nanotechnological carriers for cancer chemotherapy: the state of the art. Colloid Surface B. (2015) 126:631–48. 10.1016/j.colsurfb.2014.12.041 25591851

[32] He QJ Shi JL Mesoporous silica nanoparticle based nano drug delivery systems: synthesis, controlled drug release and delivery, pharmacokinetics and biocompatibility. J Mater Chem. (2011) 21:5845–55. 10.1039/c0jm03851b

[33] Li HR Li K Dai YP Xu XY Cao X Zeng Q. *In vivo* near infrared fluorescence imaging and dynamic quantification of pancreatic metastatic tumors using folic acid conjugated biodegradable mesoporous silica nanoparticles. Nanomed-Nanotechnol. (2018) 14:1867–77. 10.1016/j.nano.2018.04.018 29733890

[34] Liu SW Lu H Neurath AR Jiang SB. Combination of candidate microbicides cellulose acetate 1,2-benzenedicarboxylate and UC781 has synergistic and complementary effects against human immunodeficiency virus type 1 infection. Antimicrob Agents Chemother. (2005) 49:1830–6. 10.1128/AAC.49.5.1830-1836.2005 15855503 PMC1087640

[35] Alderson D Cunningham D Nankivell M Blazeby JM Griffin SM Crellin A. Neoadjuvant cisplatin and fluorouracil versus epirubicin, cisplatin, and capecitabine followed by resection in patients with oesophageal adenocarcinoma (UK MRC OE05): an open-label, randomised phase 3 trial. Lancet Oncol. (2017) 18:1249–60. 10.1016/S1470-2045(17)30447-3 28784312 PMC5585417

[36] Catenacci DVT Tebbutt NC Davidenko I Murad AM Al-Batran SE Ilson DH. Rilotumumab plus epirubicin, cisplatin, and capecitabine as first-line therapy in advanced MET-positive gastric or gastro-oesophageal junction cancer (RILOMET-1): a randomised, double-blind, placebo-controlled, phase 3 trial. Lancet Oncol. (2017) 18:1467–82. 10.1016/S1470-2045(17)30566-1 28958504 PMC5898242

[37] Lorenzen S Knorrenschild JR Haag GM Pohl M Thuss-Patience P Bassermann F. Lapatinib versus lapatinib plus capecitabine as second-line treatment in human epidermal growth factor receptor 2-amplified metastatic gastro-oesophageal cancer: a randomised phase II trial of the arbeitsgemeinschaft internistische onkologie. Eur J Cancer. (2015) 51:569–76. 10.1016/j.ejca.2015.01.059 25694417

[38] Liu J Cheng H Han L Qiang Z Zhang XW Gao W. Synergistic combination therapy of lung cancer using paclitaxel- and triptolide-coloaded lipid-polymer hybrid nanopartices. Drug Des Dev Ther. (2018) 12:3199–209. 10.2147/DDDT.S172199 30288024 PMC6161729

[39] Xu C Chen F Valdovinos HF Jiang DW Goel S Yu B. Bacteria-like mesoporous silica-coated gold nanorods for positron emission tomography and photoacoustic imaging-guided chemo-photothermal combined therapy. Biomaterials. (2018) 165:56–65. 10.1016/j.biomaterials.2018.02.043 29501970 PMC5880312

[40] Rivolta I Panariti A Lettiero B Sesana S Gasco P Gasco MR. Cellular uptake of coumarin-6 as a model drug loaded in solid lipid nanoparticles. J Physiol Pharmacol. (2011) 62:45–53. 10.0000/PMID21451209 21451209

[41] Zhu DW Tao W Zhang HL Liu G Wang T Zhang LH. Docetaxel (DTX)-loaded polydopamine-modified TPGS-PLA nanoparticles as a targeted drug delivery system for the treatment of liver cancer. Acta Biomater. (2016) 30:144–54. 10.1016/j.actbio.2015.11.031 26602819

[42] Goodison S Urquidi V Tarin D. CD44 cell adhesion molecules. Mol Pathol. (1999) 52:189–96. 10.1136/mp.52.4.189 10694938 PMC395698

[43] Almeida PV Shahbazi MA Makila E Kaasalainen M Salonen J Hirvonen J. Amine-modified hyaluronic acid-functionalized porous silicon nanoparticles for targeting breast cancer tumors. Nanoscale. (2014) 6:10377–87. 10.1039/C4NR02187H 25074521 PMC4234906

