# Supplementary material for: Construction of Biocompatible Dual-Drug Loaded Complicated Nanoparticles for in vivo Improvement of Synergistic Chemotherapy in Esophageal Cancer
Source: Front Oncol. 2020 May 5;10:622. doi: 10.3389/fonc.2020.00622 (PMC7214620; doi:10.3389/fonc.2020.00622)
Supplement: Supplementary file 1 [file Image_1.pdf]

Supplementary Material

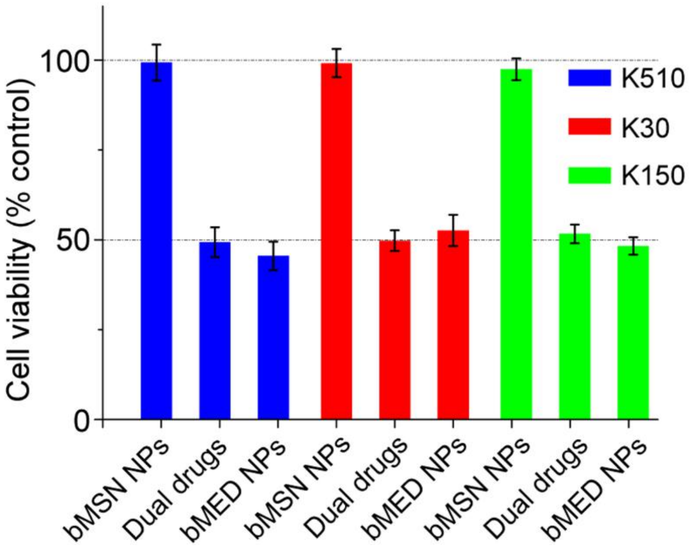

Figure S1. The cell viability of K510, K30 and K150 cells when treated with bMSN NPs, dual drugs and bMED NPs.
