# Supplementary material for: Construction of Biocompatible Dual-Drug Loaded Complicated Nanoparticles for in vivo Improvement of Synergistic Chemotherapy in Esophageal Cancer
Source: Front Oncol. 2020 May 5;10:622. doi: 10.3389/fonc.2020.00622 (PMC7214620; doi:10.3389/fonc.2020.00622)
Supplement: Supplementary file 2 [file Image_2.pdf]

Supplementary Material

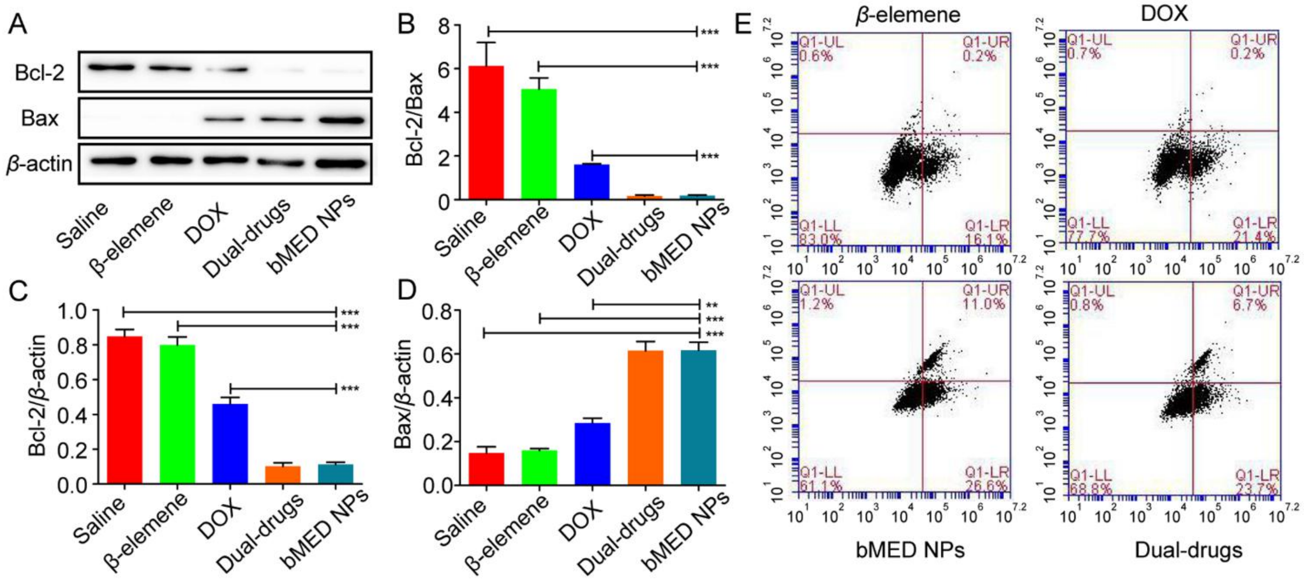

Figure S2. The western blotting results and flow cytometry results: (**A**) The expression of Bcl-2 and Bax in K30 cells when treated with saline, DOX, *β*-elemene, dual drugs, and bMED NPs for 48 hours; (**B, C, D**) The quantitative analysis of the samples. Error bars represent the SD of the mean. **p < 0.01, ***p < 0.001; (**E**) Flow cytometry analysis of DOX, *β*-elemene, dual drugs and bMED NPs.
