# Supplementary material for: Construction of Biocompatible Dual-Drug Loaded Complicated Nanoparticles for in vivo Improvement of Synergistic Chemotherapy in Esophageal Cancer
Source: Front Oncol. 2020 May 5;10:622. doi: 10.3389/fonc.2020.00622 (PMC7214620; doi:10.3389/fonc.2020.00622)
Supplement: Supplementary file 3 [file Image_3.pdf]

Supplementary Material

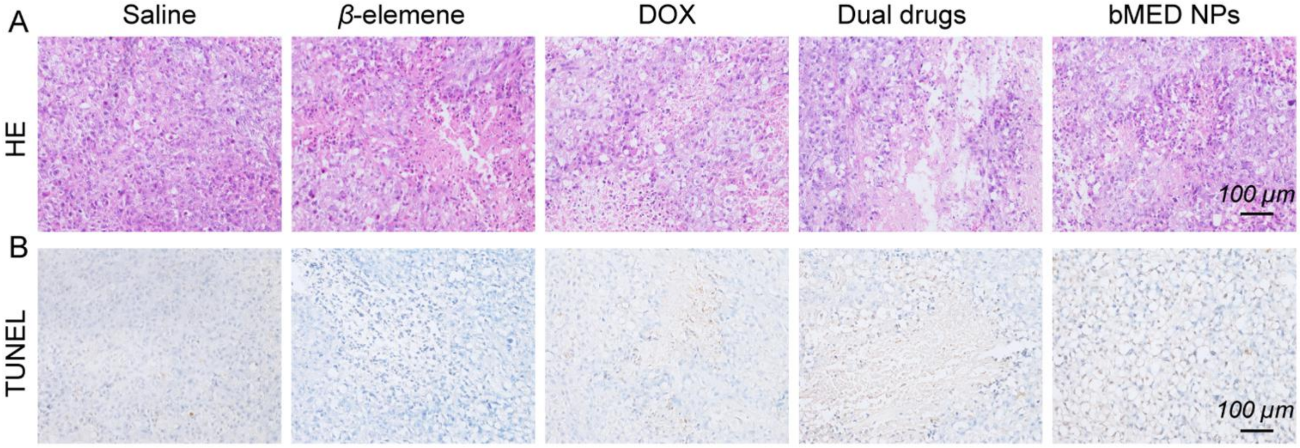

Figure S3. The mice were sacrificed and tumors were sliced and stained by H&E and TUNEL for a histological analysis.
