# Supplementary material for: Construction of Biocompatible Dual-Drug Loaded Complicated Nanoparticles for in vivo Improvement of Synergistic Chemotherapy in Esophageal Cancer
Source: Front Oncol. 2020 May 5;10:622. doi: 10.3389/fonc.2020.00622 (PMC7214620; doi:10.3389/fonc.2020.00622)
Supplement: Supplementary file 4 [file Image_4.pdf]

Supplementary Material

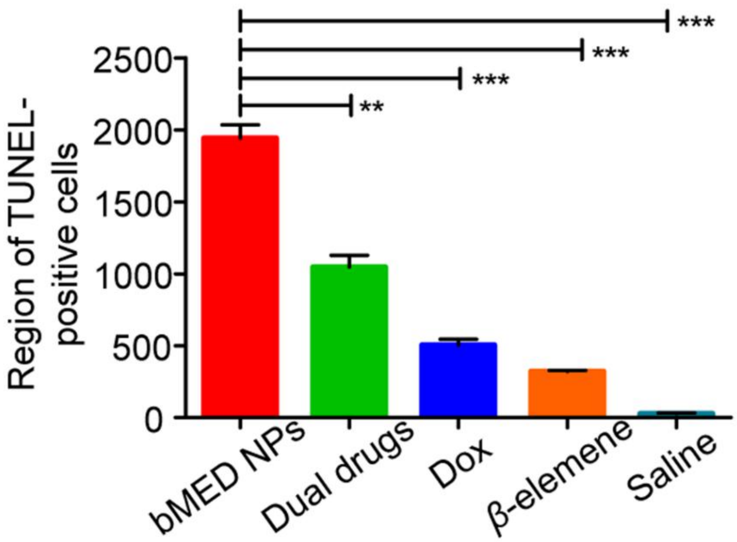

Figure S4. The Quantitative results of TUNEL staining. Error bars represent the SD of the mean. **p < 0.01, ***p < 0.001.
