# Supplementary material for: Construction of Biocompatible Dual-Drug Loaded Complicated Nanoparticles for in vivo Improvement of Synergistic Chemotherapy in Esophageal Cancer
Source: Front Oncol. 2020 May 5;10:622. doi: 10.3389/fonc.2020.00622 (PMC7214620; doi:10.3389/fonc.2020.00622)
Supplement: Supplementary file 5 [file Table_1.pdf]

Supplementary Material

Table S1. CI and dose reduction values for inhibition on K510, K30 and K150 by combining doxorubicin with β-elemene in proportion of 1:5.

% Inhibition	CI	doxorubicin			β-elemene		
		Conc.:(μM)		Dose reduction	Conc.:(μM)		Dose reduction
		Alone	Mix		Alone	Mix	
K510							
50	0.8363	27.87	8.432	3.305	355.5	43.88	8.102
75	0.9372	49.43	15.38	3.213	835.1	79.65	10.48
95	0.9882	99.87	29.48	3.388	1334	145.2	9.187
K30							
50	0.7462	38.14	12.65	3.015	478.9	65.32	7.332
75	0.7226	71.31	24.76	2.880	992.7	121.88	8.145
95	0.7521	110.3	37.13	2.971	1413	189.72	7.448
K150							
50	0.8362	31.46	10.78	2.918	297.4	52.88	5.624
75	0.7382	65.82	19.42	3.389	584.2	97.48	5.993
95	0.8632	104.5	35.97	2.905	1067	180.91	5.898
