# Supplementary material for: Construction of Biocompatible Dual-Drug Loaded Complicated Nanoparticles for in vivo Improvement of Synergistic Chemotherapy in Esophageal Cancer
Source: Front Oncol. 2020 May 5;10:622. doi: 10.3389/fonc.2020.00622 (PMC7214620; doi:10.3389/fonc.2020.00622)
Supplement: Supplementary file 6 [file Table_2.pdf]

Supplementary Material

Table S2. CI and dose reduction values for inhibition on K510, K30 and K150 by combining doxorubicin with *β*-elemene in proportion of 1:15.

% Inhibition	CI	doxorubicin			*β*-elemene		
		Conc.:(μmol/L)		Dose reduction	Conc.:(μmol/L)		Dose reduction
		Alone	Mix		Alone	Mix	
K510							
50	0.7895	27.87	4.992	5.583	355.5	75.32	4.720
75	0.8123	49.43	9.043	5.466	835.1	141.8	5.889
95	0.9124	99.87	16.46	6.067	1334	249.7	5.342
K30							
50	0.6443	38.14	7.932	4.808	478.9	117.4	4.079
75	0.6193	71.31	17.46	4.084	992.7	267.5	3.711
95	0.8322	110.3	29.36	3.757	1413	448.5	3.151
K150							
50	0.8271	31.46	6.401	4.915	297.4	95.12	3.127
75	0.8365	65.82	13.32	4.941	584.2	196.4	2.975
95	0.8993	104.5	19.35	5.401	1067	291.4	3.662
